# Acute starvation ketoacidosis in pregnancy with severe hypertriglyceridemia: A case report

**DOI:** 10.1097/MD.0000000000010609

**Published:** 2018-05-11

**Authors:** Li Hui, Li Shuying

**Affiliations:** a Department of Intensive Care Unit of Gynecology and Obstetrics; b Department of Anesthesiology, West China Second Hospital of Sichuan University, Key Laboratory of Birth Defects and Related Diseases of Women and Children, Sichuan University, Ministry of Education, Chengdu, China.

**Keywords:** acute starvation ketoacidosis, fetal distress, pregnancy, severe hypertriglyceridemia

## Abstract

**Rationale:**

Pregnant women are more prone to ketosis due to the relative insulin resistance, accelerated lipolysis and increased free fatty acids.

**Patient concerns:**

We report a pregnant woman with hyperlipidemia, who experienced severe metabolic acidosis after a short period of starvation.

**Diagnoses:**

Based on her clinical symptoms, exclusion diagnosis and therapeutic diagnosis, her condition was diagnosed as starvation ketoacidosis.

**Interventions:**

An emergency caesarean section under general anesthesia was implemented 2 hours after her admission. The metabolic acidosis was treated with fluid resuscitation using compound sodium lactate, bicarbonate, and 5% dextrose together with insulin 6U.

**Outcomes:**

Both mother and baby were discharged clinically well.

**Lessons:**

Starvation ketoacidosis may happen in special patient who was in pregnancy and with severe hypertriglyceridemia, after just one day fasting and vomiting.

## 1 Introduction

Ketoacidosis occurs most often as diabetic ketoacidosis (DKA) in type 1 diabetes mellitus, and similar ketoacidosis can also occur in patients undergoing a long fast, a condition referred to as “starvation ketoacidosis.”^[1]^ Emergency ketoacidosis during pregnancy not only has adverse effects for her mother but also for the fetus, such as neurological impairment and fetal demise. Compared with nonpregnant women, women who are pregnant are ketone-prone due to relative insulin deficiency. In 1970, Felig and Lynch^[2]^ first described this exaggerated response to fasting that results in producing more ketones during the second trimester of pregnancy. Pregnant women are more prone to ketosis due to their relative insulin resistance, accelerated lipolysis and increased free fatty acids. In this case, even a short period of starvation during pregnancy can lead to severe ketoacidosis, which is called “accelerated starvation.” In addition, pregnant women tend to develop hyperlipidemia because of their elevated levels of estrogen.

Some cases of starvation ketoacidosis occurring in pregnancy have been described previously.^[3–8]^ We report there an unusual case of starvation ketoacidosis in the third trimester of pregnancy with severe hypertriglyceridemia. The patient provided written consent and authorized us to publish her case.

## 2 Case presentation

A 37-year-old pregnant woman weighing 74 kg and 158 cm tall at 38+6 weeks of her second pregnancy was admitted with vaginal bleeding for 2 hours, Kussmaul's breathing (42/min), and history of persistent vomiting for 1 day. She had a previous history of hyperlipidemia for 2 years without any regular treatment or monitoring. She did not drink alcohol or take thiazides or B-blockers, betamethasone was not administered for fetal lung maturation, and the patient showed normal thyroid function in her second trimester examination. During this same examination, the antenatal examination indicated a slight increase in serum lipid levels (triglyceridemia: 4.92 mmol/L in her second trimester of pregnancy). On admission, the woman's body temperature, blood pressure, and peripheral oxygen saturation were normal. She appeared dehydrated, but was still fully conscious. Urinalysis revealed glucose (−), ketones (4+), and protein (1+) while random plasma glucose was 6.3 mmol/L. Findings included severe anion gap metabolic acidosis (AGMA) with pH 7.12 (normal range: 7.35–7.45); PCO_2_<10 mm Hg (normal range: 35–45 mm Hg); bicarbonate: 3 mmol/L (normal range: 21–28 mmol/L); lactate: 1.6 mmol/L (normal range: 0.7–3.0 mmol/L); and anion gap: 29.2 mmol/L (normal range: 10–18 mmol/L). Biochemical tests showed liver and kidney function were roughly normal.

The fetal biophysical score was 3, and fetal heart monitoring showed fetal distress; thus an emergency caesarean section was implemented 2 hours after admission. General anesthesia was performed, a 2610 g male baby was delivered with an APGAR score of 4 at 1 minute and 7 at 5 minutes. Umbilical cord venous pH was 6.86, pCO_2_ was 41.4 mm Hg, pO_2_ was 12 mm Hg, BE was −26 mmol/L, HCO_3_ was 4 mmol/L, and blood glucose was 1.9 mmol/L. The mother was taken to the intensive care unit (ICU) with a tracheal catheter for further treatment, her breathing pattern allowing pressure support ventilation. Her arterial blood gases (ABG) are listed in Table 1. The metabolic acidosis was treated with fluid resuscitation using compound sodium lactate and bicarbonate, but this did not correct the acidosis sufficiently. We switched this treatment to 5% dextrose, together with insulin 6U. The metabolic parameters were corrected in 24 hours, and the patient was extubated at 48 hours.

**Table 1:**
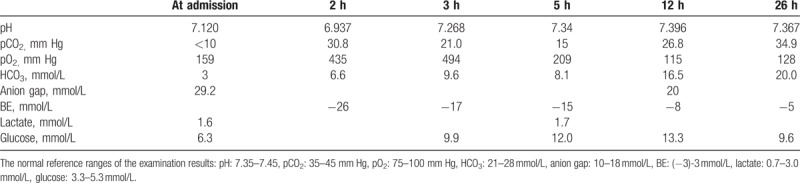
The arterial blood gases changed from hours. 2 hours: 2 hours after admission, the patient was undergoing caesarean section operation; 3 hours: 3 hours after admission, after the operation the patient was switched to ICU.

**Figure 1:**
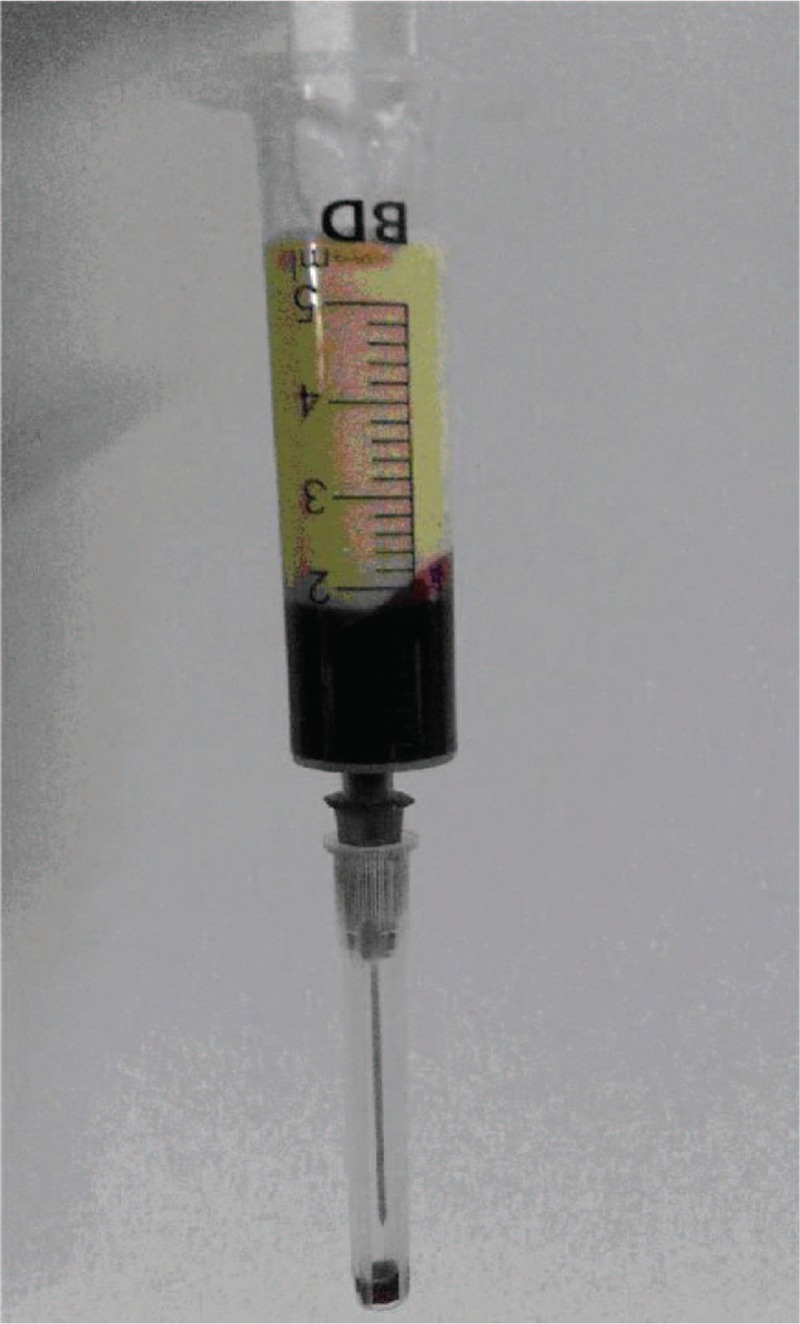
The patient's blood of chylomicronemia.

**Figure 2:**
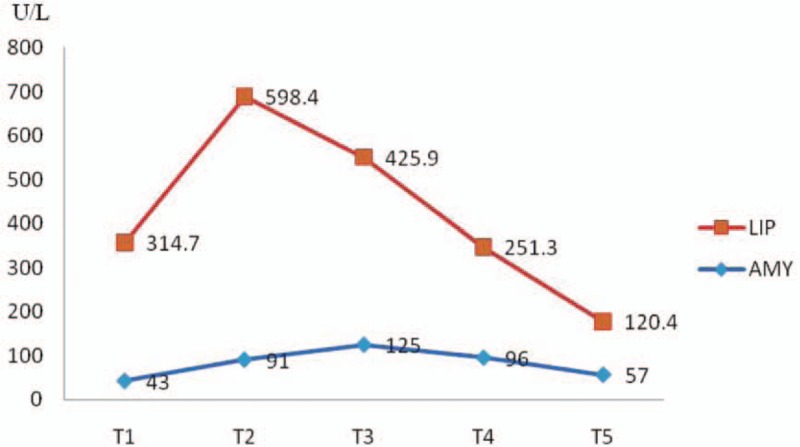
The serum levels of AMY and LIP changed from hours. *X*-axis: time (T_1_: at admission; T_2_: 6 hours; T_3_: 16 hours; T_4_: 24 hours; T_5_: 48 hours). *Y*-axis: serum levels of AMY and LIP (the normal range of AMY is 23–300 U/L, the normal range of LIP is 30–110 U/L. Both the AMY and LIP increased ≤ 3 times normal may be usually nonspecific, but when either of them elevated > 3 times normal is considered to be a highly suggestive diagnostic of acute pancreatitis). AMY = amylase, AP = acute pancreatitis, LIP = lipase.

When we performed the ABG, we found the patient's blood presented with chylomicronemia (Fig. 1). The following values were noted: serum total triglycerides (TG) were 19.93 mmol/L (normal < 2.83 mmol/L), serum total cholesterol (TC) was 12.53 mmol/L (normal < 5.18 mmol/L), high-density lipoprotein cholesterol (HDL-C) was 0.44 mmol/L (normal >1.0 mmol/L), low-density lipoprotein cholesterol (LDL-C) was 1.35 mmol/L (normal < 3.3 mmol/L), apolipoprotein A was 11.17 mmol/L (normal range: 0.76–2.14 mmol/L), and apolipoprotein B was 1.23 mmol/L (normal range: 0.46–1.42 mmol/L). We closely monitored the serum levels of amylase (AMY) and lipase (LIP) in case of acute pancreatitis (AP) (Fig. 2). An ultrasound of the abdomen was reported normal. As the hypertriglyceridemia did not induce AP, the patient chose to accept fenofibrate but not plasmapheresis to reduce the level of blood lipids. Four days later the levels of serum triglycerides declined to 11.31 mmol/L, and serum cholesterol declined to 9.86 mmol/L. At last, both the mother and baby were healthily and subsequently discharged.

## 3 Discussion

Nausea and vomiting are common symptoms in pregnancy, which could be due to hyperemesis gravidarum, acute fatty liver of pregnancy, preeclampsia, acute pancreatitis in pregnancy, metabolic acidosis, peptic ulceration, and gastroenteritis. With the loss of gastrointestinal contents and inadequate intake, it can lead to acid–base disturbance, especially in pregnant woman. After a lengthy fast, hepatic glycogen stores are quickly depleted, and lipid mobilization is enhanced, thus resulting in ketone body generation and acidosis. This is also the formation process of starvation ketoacidosis.

There are just a few blood ketone bodies in healthy individuals after fasting or prolonged exercise. Starvation ketoacidosis in the nonpregnant individual is very rare in developed countries. It requires at least 14 days to reach mild ketoacidosis with pH usually above 7.3.^[9,10]^ Several hormones generated in pregnancy, including progesterone, cortisol, and human placental lactogen, can contribute to a diabetogenic state presenting as insulin resistance, increased lipolysis, augmented free fatty acids and ketogenesis.^[11]^ Maternal ketone body levels increase in gestation 2–3-fold from baseline, and the relative insulin resistance also gradually increases with gestational time, resulting in susceptibility to a ketotic state.^[12]^ The accelerated starvation that increases the tendency to ketosis has been described in the second and third trimesters.^[2,13,14]^ In addition, hyperventilation and reduction in PCO_2_ caused by progesterone and lung volume changes can lead to chronic respiratory alkalosis, which, in turn decreases the plasma bicarbonate concentration, thus leading to an increased tendency toward a ketotic state in the third trimester of pregnancy.^[8]^

The plasma levels of all kinds of lipoproteins, including cholesterol, triglycerides, very low-density lipoprotein cholesterol, LDL-C, and HDL-C increase physiologically during pregnancy. They may show a mild increase of 2–3-fold from prepregnancy levels due to the elevated levels of estrogen.^[15–17]^ Such increases are usually not very severe and can be well tolerated; however, when the patient has pre-existing abnormal lipid metabolism, gestational hypertriglyceridemia can be exacerbated.^[18]^ The Endocrine Society recommends that hypertriglyceridemia can be classified into mild (150–199 mg/dL or 1.7–2.3 mmol/L), moderate (200–999 mg/dL or 2.3–11.3 mmol/L), severe (1000–1999 mg/dL or 11.3–22.6 mmol/L), and very severe (>2000 mg/dL or >22.6 mmol/L). Mild or moderate hypertriglyceridemia may aid in evaluating the risk of cardiovascular disease, whereas severe and very severe hypertriglyceridemia could be responsible for pancreatitis.^[19]^ Gestational hypertriglyceridemia may enhance lipolysis and increase the free fatty acids, thus boosting ketogenesis.

The common reasons for metabolic acidosis with a high anion gap include ketoacidosis (possibly attributable to having diabetes, euglycemic diabetes, or chronic alcoholism or experiencing starvation) and lactic acidosis.^[3]^ The anion gap is a calculated measure (anion gap = (Na + K)–(Cl + HCO_3_), normal range: 12–16 mol/L), and it can help elucidate the cause of metabolic acidosis. The patient did not have a history of excessive alcohol consumption, so alcoholic ketoacidosis (AKA) was eliminated. We also excluded lactic acidosis, because the serum lactate level in the woman was normal. As the random plasma glucose and the glucose tolerance tests were in the normal range, the diagnosis of diabetic ketoacidosis (DKA) and euglycemic diabetic ketoacidosis were unlikely. We made the diagnosis of starvation ketoacidosis in this woman based on the exclusion of other causes, her history of one day of vomiting caused by acute gastroenteritis, the positive presence of ketonuria, severe AGMA and metabolic acidosis, which was improved quickly by 5% dextrose but not sodium bicarbonate and intravenous fluids. The critical management for this type of patient was early identification the pathogenesis, and the optimal therapeutic approach for starvation ketoacidosis was carbohydrate administration. In this case, we did not recognize the pathogenesis at first, and we managed initially with sodium bicarbonate and intravenous fluids, which did not improve the crisis acid-base disturbance.

As the diagnosis of acute pancreatitis was not established, the women rejected therapeutic plasmapheresis when fenofibrate was used to decrease the serum level of triglyceride. There are some reports suggesting that triglyceride levels are reduced significantly after delivery within 24 hours.^[20,21]^ Although severe hypertriglyceridemia may lead to acute pancreatitis and chylomicronemia, some pregnant women can easily tolerate this extremely high level of triglyceride without any significant symptoms and complications.

## 4 Conclusions

Our case indicated that fasting and hypertriglycemia should be strictly avoided during late pregnancy when starvation ketoacidosis could be induced after even short-term fasting, especially in those with severe hypertriglyceridemia. However, in patients with severe hypertriglyceridemia who can successfully toletate, supplementation of carbohydrates, this is an important course rather than sodium bicarbonates or plasmapheresis.

## Author contributions

**Data curation:** Li Hui, Li Shuying.

**Investigation:** Li Shuying.

**Methodology:** Li Hui.

**Supervision:** Li Shuying.

**Writing – original draft:** Li Hui.

**Writing – review & editing:** Li Hui.
